# Adhesive Interactions Delineate the Topography of the Immune Synapse

**DOI:** 10.3389/fcell.2018.00149

**Published:** 2018-10-30

**Authors:** Noa Beatriz Martín-Cófreces, Miguel Vicente-Manzanares, Francisco Sánchez-Madrid

**Affiliations:** ^1^Servicio de Inmunología, Instituto de Investigación Sanitaria Princesa (IP), Hospital Universitario de la Princesa, Universidad Autónoma de Madrid, Madrid, Spain; ^2^Centro de Investigación Biomédica en Red de Enfermedades Cardiovasculares (CIBERCV), Madrid, Spain; ^3^Centro de Investigación del Cáncer-Instituto de Biología Molecular y Celular del Cáncer, CIC-IBMCC (CSIC-Universidad de Salamanca), Salamanca, Spain

**Keywords:** immune synapse, cytoskeleton, actin, T cell receptor, integrin, LFA-1, VLA-4

## Abstract

T cells form adhesive contacts with antigen-presenting cells (APCs) as part of the normal surveillance process that occurs in lymph nodes and other tissues. Most of these adhesive interactions are formed by integrins that interact with ligands expressed on the surface of the APC. The interactive strength of integrins depends on their degree of membrane proximity as well as intracellular signals that dictate the conformation of the integrin. Integrins appear in different conformations that endow them with different affinities for their ligand(s). Integrin conformation and thus adhesive strength between the T cell and the APC is tuned by intracellular signals that are turned on by ligation of the T cell receptor (TCR) and chemokine receptors. During the different stages of the process, integrins, the TCR and chemokine receptors may be interconnected by the actin cytoskeleton underneath the plasma membrane, forming a chemical and physical network that facilitates the spatiotemporal dynamics, positioning, and function of these receptors and supports cell-cell adhesion during T cell activation, allowing it to perform its effector function.

## Introduction

Cell adhesion is the basis of multicellular organism coherence. Adhesive strength determines the intrinsic stability of tissues and organs. The immune system is formed partly by autonomous, motile cells that interact with each other or with non-immune cells through transient adhesive contacts. The onset of these contacts during development establishes self-recognition as well as pathogen detection. T cells can recognize specific antigens in the context of the major histocompatibility complex I or II (MHCI or II) through their T cell receptor (TCR) (Davis et al., 1998). The contact with antigen-presenting cells (APCs) relies on antigens, but also on non-antigenic, adhesive contacts (Dustin and Springer, 1989). Unlike epithelial monolayers, in which cadherin-type molecules mediate the majority of cell-cell contacts, T cell adhesive interaction with APCs is mainly driven by integrin receptors and their ligands. Integrins are also relevant for migration and adhesion of immune cells in other contexts. Integrins are heterodimeric transmembrane proteins formed by an alpha (α) and a beta (β) chain. Classical lymphocyte integrins include αLβ2 (also known as LFA-1 or CD11a/CD18), αMβ2 (Mac-1 or CD11b/CD18), αXβ2 (p150,95 or CD11c/CD18), and α4β1 (VLA-4 or CD49d/CD29) (Vicente-Manzanares and Sanchez-Madrid, 2004). Integrins regulate their adhesive ability through two major mechanisms: its intrinsic affinity for an individual ligand, which depends on its conformation; and its collective adhesiveness (“avidity”), which is regulated by its aggregation state (degree of clustering) at the plasma membrane. Through these processes, integrins fine-tune cell adhesion; likewise, deadhesion, or detachment is dependent on integrin internalization, i.e., their removal from the plasma membrane, and by binding to molecules that decrease their affinity and/or avidity (Vicente-Manzanares et al., 2009). Cycles of adhesion and detachment from neighbor surfaces allow cell movement on substrates and transient cell-cell interactions. Immune cells are swift migrators; they move at speeds of 5–40 μm/min, contrasting with fibroblasts (0.1–1 μm/min) (Smith et al., 2003), which allows their rapid recruitment; e.g., to inflammatory foci. Leukocyte Adhesion Deficiency (LAD-I), a rare recessive autosomal primary immunodeficiency, points out the relevance of β2 integrins. The main clinical features of LAD are persistent leukocytosis, recurrent life-threatening infections, impaired pus production, and reduced wound healing. Patients suffering from LAD have impaired cell surface expression of β2 heterodimers such as LFA-1, Mac-1, and p150/95, leading to defective leukocyte adhesion to endothelial cells, absence of diapedesis and substandard phagocytosis (Anderson and Springer, 1987). In LAD-3, *kindlin3*, encoding an integrin-actin connector for hematopoietic cells, is mutated, and patients show a phenotype resembling LAD-1 and thrombasthenia-like bleeding problems due to defective leukocyte and platelet adhesion (Svensson et al., 2009). Interestingly, other aspects of lymphocyte tissue navigation, e.g., through the lymph nodes, does not seem to require typical integrins; in particular, T lymphocytes from *Itgb2*^−/−^ and *talin*^−/−^ mice migrate similar to wild-type T cells (Woolf et al., 2007; Lammermann et al., 2008). Also, T cell egress from lymph nodes through cortical sinus vessels into the efferent lymph is independent of LFA-1 and VLA-4 (Arnold et al., 2004). The presence of different integrins in T cells, such as α4β7 or αvβ3, might compensate the deficiency in *Itgb2, Itgal*, or *Itga4* in specific contexts (Walling and Kim, 2018).

Integrins also act as signal transducers in both directions, extracellular, and intracellular. Outside-in and inside-out signaling influence the conformation of the integrins, depending on whether the modulating factors are extracellular or intracellular (e.g., binding to their ligands or binding of actin-connector talin to its intracellular tail, respectively Tadokoro et al., 2003). The contact of T cells with an antigen-presenting cell and signaling through the TCR deliver a stop signal that enables the formation of the immunological synapse (Dustin et al., 1997). Migratory arrest requires talin, which recruits vinculin and F-actin to the integrin cytosolic tail at the T-APC plasma membrane contacting sites, stabilizing the interaction (Wernimont et al., 2011). During the formation of the immunological synapse, adhesion enables a proper scanning of the APC surface by the T cell (Montoya et al., 2002; Martin-Cofreces et al., 2014) to allow the TCR-dependent activation of the T cell (Frauwirth and Thompson, 2002). More recently, the independence from actin cytoskeleton for initial TCR-pMHC contacts mediated by TCR localized in microvilli has been reported (Cai et al., 2017). In this review, we will discuss the crosstalk between integrins, TCR and chemokine receptors through intracellular second messengers that influence T-APC adhesion during immune synapse formation.

## LFA-1 and calcium fluctuations in the immune synapse

**Figure 1:**
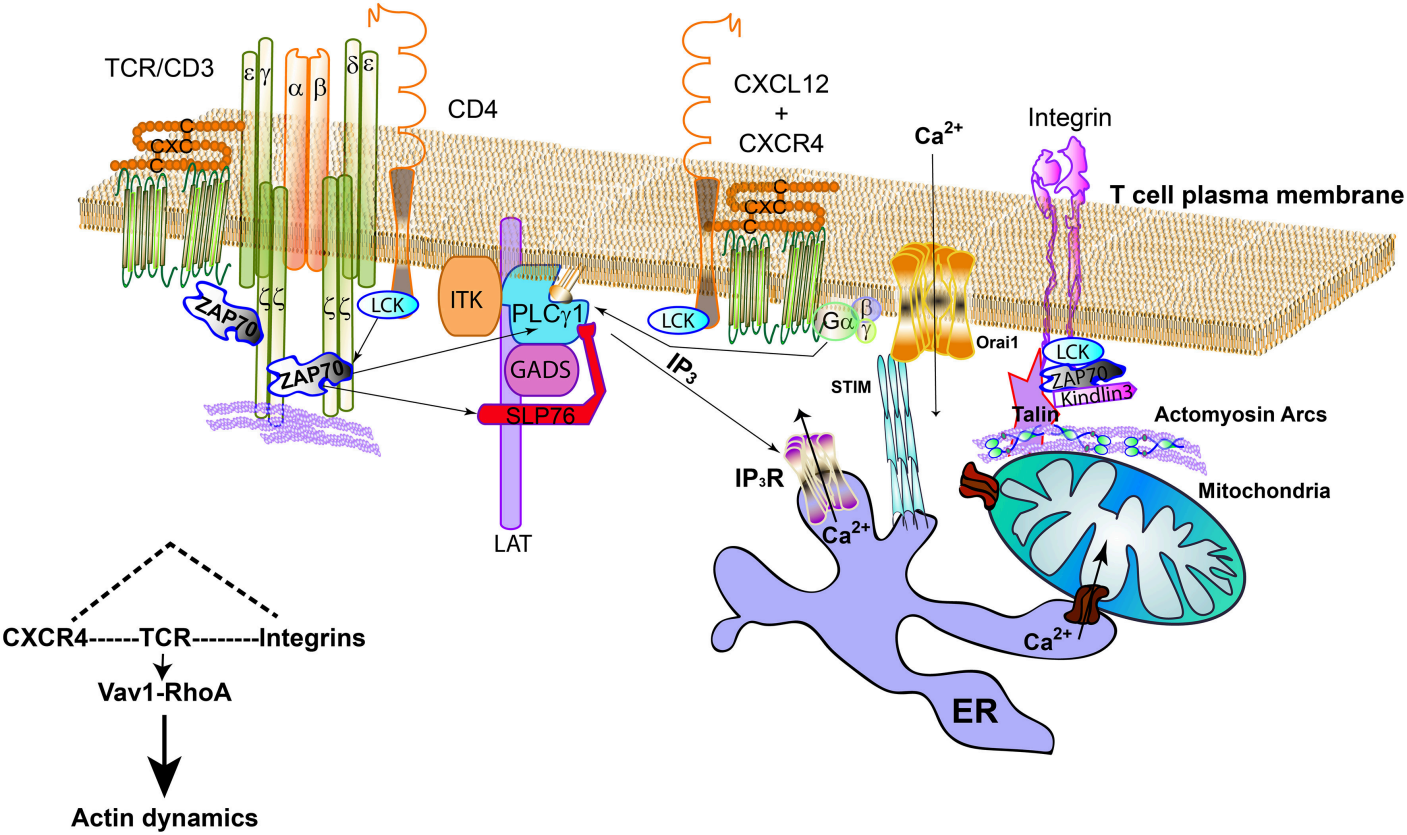
TCR, LFA-1, and CXCR4 connecting signalosomes at the IS. The TCR activates by the binding to cognate antigen-MHC complexes; in CD4 T cells, Lck bound to CD4 can help the phosphorylation of the cytoplasmic tails of CD3 subunits and ZAP70. CXCR4/CD4 complexes are found at the plasma membrane. Indeed, it can also interact with the TCR. ZAP70 is part of the CXCR4/TCR complex. CXCR4 may act on PLCγ1, regardless binding to LAT, through the G proteins or ZAP70, producing inositol-3-phosphate (IP_3_), which will activate the specific receptor (IP_3_R) at the endoplasmic reticulum (ER). This receptor allows Ca^2+^ flowing from internal stores. The mitochondria can dock at the immune synapse and form specific bonds to the ER to facilitate the buffering of Ca^2+^. The activation of STIM at the ER promotes its clustering and the activation of CRAC channels (Orai1) at the plasma membrane, increasing the intracellular Ca^2+^ by channel opening. Integrins cooperate with specific signaling elements such as Lck and ZAP70 to activate Kidling3 and Talin, allowing the binding to actin and actomyosin arcs. TCR, CXCR4, and integrins signaling pathways share the Vav1-RhoA axis.

Calcium is a non-synthesized and highly diffusible, very-early second messenger in T cells, playing an essential role during the initial steps of IS formation. It influences signal transduction, cell reorganization and nuclear activation (Fracchia et al., 2013; Martin-Cofreces et al., 2014). The interaction with APCs bearing antigenic pMHC provokes a quick increase of cytosolic [Ca^2+^]; when co-stimulation is absent during activation with high-affinity antigenic peptides, T cells make short-lived contacts with APCs and exhibit weak and infrequent Ca^2+^ spikes (Wei et al., 2007). T lymphocytes increase their intracellular calcium levels through the action of PLC enzymes upon TCR activation, chemokine receptor ligation and co-stimulation, e.g., CD28 (Feske, 2007). PLCγ1 hydrolyzes PIP_2_ (phosphatidylinositol-3,4-bisphosphate) to IP_3_ (inositol-1,4,5-trisphosphate) and DAG (diacylglycerol). The binding of IP_3_ to its receptor (IP_3_R) in the endoplasmic reticulum (ER) membrane causes the release of the Ca^2+^ stored in the ER (Figure 1). T cells also express membrane-bound calcium channels encoded by the *Orai* genes. The hexameric channels formed by Orai subunits (Hou et al., 2012) become open upon activation of STIM1 and 2 in the ER, leading to aggregation of STIMs at the ER membrane. STIM1 activation depends on calcium release from the ER (Liou et al., 2005; Roos et al., 2005). Orai/STIMs are known as calcium-release calcium channels (CRACs). Although CD4 T cells from *Orai*^−/−^ show normal proliferation rates but low activation-induced cell death (Kim et al., 2011), Orai2 and Orai3 expression may account for normal calcium fluxes. In this regard, CTLA-4 activation, which accounts for T-DC late detachment events after TCR activation, inhibits CRAC channel activation, possibly through Orai3 and STIM2 (Thiel et al., 2010). This may represent another mechanism by which CTLA-4 shuts off TCR signaling. CTLA-4 has been described to bind to and decrease the number of CD80/CD86 molecules available to CD28 by trans-endocytosis at the T cell (Qureshi et al., 2011) and to displace CD28 from the central area of the IS (Yokosuka et al., 2010). Caveolin-1, a transmembrane protein regulating membrane curvature, controls the activity of CRAC channels and the downstream activation of c-fos and NFAT through mechanisms dependent and independent of caveolin-1 phosphorylation at Y14 residue, respectively (Yeh and Parekh, 2015). Caveolin-1 interacts with Orai1, either directly or indirectly (Yu et al., 2010). A likely scenario involves LFA-1 activation and its position at the IS intertwined with Ca^2+^ flow during T cell activation through caveolin-1. CRAC channels connect to LFA-1 in neutrophils, as Orai1 interacts with kindlin3, and help LFA-1 to adopt a high-affinity conformation at LFA-1 clusters (Dixit et al., 2012). This is relevant because CRAC components localize at the IS of T cells inside the actin ring, correlating with the accumulation of ER tubules. In this context, it was proposed that the flux of calcium increased actin depolymerization (for example through the action of Ca^2+^-sensitive actin severing proteins, e.g., gelsolin), thereby decreasing F-actin concentration at the central area of the IS and confining actin polymerization to the more distal areas (Hartzell et al., 2016).

Mitochondria are involved in early T cell activation by tethering to the ER through mitofusin 2 and acting as local buffers of calcium (Lioudyno et al., 2008; Quintana et al., 2011). Mitochondria translocate to the IS during its formation, enabling the occurrence of ER-mitochondria contacts (Quintana et al., 2007). At this location, they exert local control of calcium concentration through CRAC channels in an actin and tubulin- and mitochondrial fusion and fission-dependent manner (Baixauli et al., 2011; Maccari et al., 2016). Mitochondrial delocalization during IS formation leads to a defective activation of canonical signaling events downstream of calcium elevation, e.g., myosin II phosphorylation (Baixauli et al., 2011), even if global calcium is increased in the activated T cell (Baixauli et al., 2011; Quintana et al., 2011).

Chemokine activation and LFA-1 binding to its ligands can re-localize mitochondria in T cells to some extent in the absence of TCR activation (Contento et al., 2010), priming this signaling pathway prior to full TCR engagement. CXCR4 is a chemokine receptor that belongs to the family of G protein-coupled, 7-transmembrane receptors. Among many functions, these receptors drive lymphocyte migration and integrin inside-out activation. CXCR4 associates with CD4, which in turn binds to Lck. Its ligand, CXCL12, acts as chemoattractant that is involved in the mobilization of hematopoietic progenitors and also controls leukocyte recirculation in inflammatory and homeostatic conditions (Zlotnik et al., 1999). CXCR4 accumulates at the immune synapse, where it interacts with the TCR, enhancing Erk1/2 signaling through the activation of ZAP70 (Kumar et al., 2006). ZAP70 phosphorylates the adaptor LAT (Zhang et al., 1998), which enables the docking of PLCγ1 through SH2 domains (Lin and Weiss, 2001). CXCR4 localizes with integrins in the same general region at stable IS, forming part of the adhesive external ring (Perez-Martinez et al., 2010). There, it increases the recruitment of talin, thereby facilitating the conformational extension of LFA-1 and cell adhesion upon binding to its ligand at the APC. It also boosts signaling by Jak1 and 2 and Gi (Cascio et al., 2015), which also contributes to LFA-1 activation. Specifically, RhoA activation mediated by Jak2 and 3 in collaboration with the GEF Vav1 increases LFA-1 activation during lymphocyte migration (Montresor et al., 2013), and a similar role can be inferred at the IS. These data support the role of chemokines in the formation of the IS by localizing and activating integrins, resembling the process of extravasation to tissues. In this context, chemokines on the glycocalix of the blood vessel wall trigger the inside-out priming of leukocyte integrins, which mediates firm adhesion and allows cell polarization (del Pozo et al., 1995). It is important to note that some of these signaling modules, e.g., Lck-ZAP70 and Vav1-Rho are shared by chemokine receptors and the TCR, becoming cross-roads that lead to T cell activation and calcium mobilization. A relevant question is how Lck and ZAP70 meet at LAT sites to allow PLCγ1 recruitment and activation. Lck cannot phosphorylate LAT, but ZAP70 does (Zhang et al., 1998), and ZAP70 binds to the TCR/CD3 complex upon phosphorylation by Lck, forming different signalosomes (Martin-Cofreces et al., 2014). In this regard, a recent report described the direct binding of Lck and LAT through the SH3 domain of Lck to a Pro-rich region near the transmembrane region of LAT, bringing ZAP70 into close proximity to LAT through binding of Lck SH2 domain to ZAP70. The poly-Pro sequence in LAT is essential for Ca^2+^ influx upon TCR activation (Lo et al., 2018). These observations agree with the observed formation of early micro-arrays of TCR and LFA-1 at the IS, with relevant proteins for F-actin binding, such as paxillin, forming adhesive structures that facilitate LAT recruitment (Hashimoto-Tane et al., 2016). They are also in agreement with the observation that LAT and TCR localized at different “protein-islands” that join upon activation (Lillemeier et al., 2010). Therefore, LFA-1, CXCR4, and the TCR may cooperate with each other through the recruitment of LAT due to the bridging activity of Lck, thus increasing Ca^2+^ influx and facilitating integrin activation.

## Integrins in the IS: the actin connection

Integrins form specialized adhesive structures in mesenchymal cells. These include focal complexes, focal and fibrillar adhesions. These structures serve as convergence points between the extracellular matrix and the actin cytoskeleton. They contain a large number of scaffolding and signaling molecules. T cells only form small complexes in which integrins interact with different actin-binding proteins, e.g., talin, α-actinin, filamin A, and ILK (Vicente-Manzanares and Sanchez-Madrid, 2004). As depicted above, these small-sized adhesive structures may form early during IS formation (Hashimoto-Tane et al., 2016), in a “fractal” mode to achieve enlarged assemblies upon IS maturation, probably depending on the stability and duration of the T-APC contact. Filamentous (F-) actin adopts different architectures, from long bundles to reticulate networks, endowing the cell with functional plasticity. In lymphocytes, the majority of F-actin appears immediately under the plasma membrane, forming the cortical cytoskeleton, also known as actin cortex. During the initial contacts of the T cell with the APC, the interaction of LFA-1 with ICAM-1 triggers the phosphorylation of the beta2 integrin chain, initiating co-stimulatory T cell signaling events that induce cytoskeleton rearrangements and nuclear activation (Perez et al., 2003; Nurmi et al., 2007). In this context, T cells extend actin-based microvilli that facilitate TCR-pMHC interaction events. Microvilli appearance is independent of TCR signals, but successful TCR engagement stabilizes them. Once productive interactions are established, actin polymerization facilitates the spreading of the T cell over the APC (Beemiller et al., 2012; Cai et al., 2017). The stabilization of the contact interface region between the two cells allows the actin polymerization at the external part of the lamella to establish a retrograde flow underneath the plasma membrane to further regulate the positioning of the proteins linked to the actin cytoskeleton, either at intracellular locations or at the plasma membrane. These events underlie a finely controlled distribution of molecular components in different regions along the T: APC interface during IS maturation, forming “supra molecular activation clusters” (SMACs). This was one of the earliest identified features of the IS (Monks et al., 1998; Grakoui et al., 1999). Upon the establishment of high-affinity interactions between the TCR and the MHC: antigen complex, SMACs form within minutes. Actin is a major player in the molecular segregation in different SMACs, through its ability to generate a centripetal flux of actin-bound molecules from the periphery of the contact [distal and peripheral SMACs; dSMAC; and pSMAC) to the central area (cSMAC), conforming a “bullseye” organization (Bunnell et al., 2001; Varma et al., 2006; Kaizuka et al., 2007; Babich et al., 2012; Smoligovets et al., 2012). It is of note that not all the synapses formed by T cells and APCs display “bullseye”-shaped SMACs, but instead there is a great variability in shape and structure depending on the cell types involved, the strength of antigen recognition, if present, and additional co-stimulatory interactions (Friedl et al., 2005; Azar et al., 2010; Thauland and Parker, 2010). In T-DC contacts, the synapses are often different from the classical IS described as concentric SMACs, although a similar structure has been described for *in vitro* bone-marrow derived DCs, both conventional DCs and FLT3L-derived plasmacytoid DCs (Mittelbrunn et al., 2009). Multiphoton imaging from lymph node explants and intravital imaging in live mice have been used to analyze T-DCs contacts. Short interactions are detected in absence of antigen (< 3 min; Miller et al., 2004a), allowing thousands of scans on migrating T cells (Miller et al., 2004b). The contact with different DCs is extended upon recognition of the antigen (Dustin et al., 1997; Friedl et al., 2005); DCs may contact several T cells simultaneously. The availability of the antigen and the number of antigen-presenting DCs determine the ratio of DC:T cells forming contacts (Henrickson et al., 2013). The duration of these stable, long-lived T-DC contacts has been estimated to be about 3–5 h, with a detachment stage that reestablishes T cell motility and proliferation after this phase (Hommel and Kyewski, 2003; Mempel et al., 2004; Beltman et al., 2009). Short-lived contacts are sufficient for T cell activation, corresponding with *in vitro* reports showing that activation of helper T cells by DCs is observed upon short and sequential interactions (Hommel and Kyewski, 2003; Mempel et al., 2004). These interactions do not allow complete formation of the SMACs due to spatiotemporal restrictions, and probably by mechanical counter forces from the DC preventing TCR clustering at the central area of the contact (Friedl et al., 2005). When a long-term immune synapse is established, the organization and movement of TCR microclusters is chiefly directed by actin toward the center of the contact (Kaizuka et al., 2007; DeMond et al., 2008; Mittelbrunn et al., 2009). Polymerized actin at the dSMAC fuels the formation of actomyosin arcs that co-localize to LFA-1 and allow TCR movement through the pSMAC (Murugesan et al., 2016). An intracellular partner of caveolin-1 is endothelial nitric oxide synthase (eNOS) (Garcia-Cardena et al., 1997), which is post-translationally palmitoylated and bound to caveolin-1 when inactive. In resting T cells, eNOS localizes at the Golgi and regulates TCR activation upon translocation to the plasma membrane in a calcium- and phosphoisoitide-3-kinase (PI3K)-dependent manner (Ibiza et al., 2006). The Golgi apparatus appears polarized toward the IS together with the centrosome upon TCR activation (Martin-Cofreces et al., 2014). At the IS, eNOS regulates the post-translational modification of actin through nitrosylation to promote actin depolymerization at the cSMAC and to allow the correct flow of actin (Garcia-Ortiz et al., 2017). Also, the RhoA-ROCK axis can activate eNOS, which could regulate the production of nitric oxide at the IS (Amano et al., 2010) and link eNOS activity to the TCR-CXCR4-LFA-1 axis. It is feasible that eNOS localizes to caveolin-1 islets at the plasma membrane of T cells upon activation, facilitating eNOS proximity to CRAC channels and LFA-1. At this location, eNOS can mediate the proper distribution of the cSMAC and pSMAC components by controlling actin dynamics.

Interestingly, TCR dynamics in the external area of the IS correlates well with the dynamics of concentric arcs of non-muscle myosin IIA (NMII-A) (Kaizuka et al., 2007; Murugesan et al., 2016). The presence of NMII-A mainly at the pSMAC (but barely at the cSMAC) seems to determine the different actin flow rates at dSMAC and pSMAC (Yi et al., 2012), similar to the differences in flow rates observed at the lamellipodium and the lamellum of mesenchymal cells (Ponti et al., 2004). At the p-SMAC, actomyosin arcs co-localize with LFA-1 clusters (Murugesan et al., 2016), which correlates well with the known function of NMII-A as a major regulator of integrin clustering in other cell types (Vicente-Manzanares et al., 2009). Although NMII-A is required for lymphocyte migration and the onset of the stop signal during the formation of the IS (Jacobelli et al., 2004), its role at the IS seems controversial. In this regard, one study suggested that NMII-A controls TCR congregation at the cSMAC (Ilani et al., 2009), which is likely due to a positive regulation of the phosphorylation of the regulatory light chain helped by localization of mitochondria at the IS (Baixauli et al., 2011). Another study indicated that NMII-A collaborates with dynein to regulate the translocation of the centrosome to the IS, but acting at a distance (Liu et al., 2013). Finally, an additional study found no role for NMII-A in IS formation (Jacobelli et al., 2004). However, it is possible that these differences are due to the expression of different molecular partners that vary across cell types or species.

The connection of integrins to actin is important for their activation. Many scaffold proteins play fundamental roles in these interactions. For example, LFA-1 activation during migration depends on scaffold proteins such as AKAP450 (El Din El Homasany et al., 2005). At the IS synapse, the high affinity conformation of LFA-1 has been observed using conformation-sensitive antibodies, such as Kim127 (extended conformation, intermediate-high affinity) and m24 (extended-open, high affinity conformation). This activation pattern was disrupted by inhibition of actin flow (Comrie et al., 2015a) and the inhibition of AKAP450 during IS formation, which was accompanied by a lack of sustained activation of signaling emanating from the TCR/CD3 complex, including the phosphorylation of CD3ζ, PLCγ1, and PKCθ (Robles-Valero et al., 2010). Conversely, accumulation of diacylglylicerol at the IS was increased, which could reflect the accumulation of activated integrins at the cSMAC (Bustos-Moran et al., 2017).

## Reciprocal reorganization at the T cell and antigen-presenting cell

The adhesive and signaling molecules located at the interface of T cells and APCs favor intercellular adhesion, allowing the exchange of information. There are also molecular partners facilitating detachment, which is relevant for immune contacts with different cells. This has been known for a while in the case of T cells, but it is a new field in the case of APCs. Several studies have described the reorganization of DCs during immune synapse formation, including the clustering of MHCII and ICAM-1. ICAM-1 and −3 mobility in DCs mirrors LFA-1 clustering in T cells, allowing the concentration of MHC-II at the DC surface in contact with the T cell (de la Fuente et al., 2005). This enhances antigen sampling by the TCR and the subsequent formation of microclusters. More recently, the clustering of ICAM-1 at the DC was found upon LFA-1 binding, but not that of MHC-II. Drugs disrupting the actin cytoskeleton mainly abrogated co-localization of MHC-II and ICAM-1 at the DC, reducing MHC-II mobility while increasing ICAM-1 mobility (Comrie et al., 2015b). A relevant question is whether LFA-1 at the DC may be active or not, also regulating interaction with the T cell. The expression of ICAM-1 in T lymphocytes, mainly memory and effector T cells, has a clear influence in T-DC conjugates. LFA-1 appears to be inactive in DCs, but increasing its activation may extend the duration of the interaction with CD4 and CD8 T cells, leading to decreased T cell activation and proliferation (Balkow et al., 2010).

Different molecules can affect integrin or ICAM-1 organization through lateral interactions. One example are tetraspanins, expressed by both T cells and APCs (Saiz et al., 2018). Caveolae-regulating proteins, e.g., caveolin-1, may also be important either on the T cell side or on the APC side, although caveolae are not present in T lymphocytes. CD8 T cells from *Cav1*^−/−^ mouse respond poorly to antigen, displaying deficient IS formation, low IFNγ production and clonal expansion when challenged with *L. monocytogenes* (Tomassian et al., 2011). At a molecular level, *Cav1*^−/−^ CD8 T cells could not recruit LFA-1 to the cytolytic synapse and displayed reduced interaction regions with the target cell. This defect was due to a deficient response via TCR/CD3, as LFA-1 affinity for ICAM-1 was unaffected upon TCR activation, in contrast to the lower clustering of the integrin (Borger et al., 2017), corresponding with the defective recruitment of LFA-1. In CD4 T cells, caveolin-1 plays a structural role and is needed for the optimal clustering of Lck and the TCR; T cell grafts from wild type and *Cav1*^−/−^ mice are equally potent at inducing graft vs. host disease (GVHD) when depleted of regulatory T cells (Tregs), indicating that *Cav1*^−/−^ Tregs are equally protective (Schonle et al., 2016). Caveolin-1 increases in DCs upon their maturation and facilitates their migration to the lymph node and the chemotaxis in response to CCL21. *Cav1*^−/−^DCs failed in forming membrane protrusions in response to LPS and in activating Rac-1. Indeed, the ability to eliminate B16F10 tumors, a model for melanoma, *in vivo* upon vaccination with wild-type or *Cav1*^−/−^ DCs decreases in the latter, pointing to defects in CCR7 chemokine receptor and probably integrin activation (Oyarce et al., 2017). In this sense, CCR7 bearing CD103^+^/CD141^+^ DCs are crucial for activating T cells inside tumor to increase melanoma rejection (Roberts et al., 2016). These CD103+ DCs are also relevant for recruitment of effector T cells to inflamed melanoma model through CXCL10 production (Spranger et al., 2017). Production of CXCL10 by DCs upon IS formation with T cells has been recently reported to be dependent on mitochondrial DNA transfer through exosomes and activation of the STING pathway (Torralba et al., 2018). Since the STING pathway for cytosolic DNA sensing mediates recognition of immunogenic tumors (Woo et al., 2014), it is conceivable that the stability of immune synapses established inside the tumor by T cells may increase the activity of CD103+DCs in recruiting T cells through CXCL10 production, likely forming a feedback loop. A recent report has shown that NK cells interacting with CD103+DCs in the tumor microenvironment support their presence in the tumor; NK cells form further and more stable interactions with DCs than T cells and seem to increase DC survival (Barry et al., 2018). The establishment of synapses with T cells also seems to improve DC survival either *in vivo* or *in vitro* by avoiding apoptosis events; CD40 activation was relevant for this process (Riol-Blanco et al., 2009). Therefore, stable synapses should improve the response to different threats.

CD103 is the integrin αE (mainly representing αEβ7), a receptor for E-cadherin that enables T cells and DCs to colonize the skin and intestinal mucosa, by supporting their interaction with epithelia (Cepek et al., 1994; Pauls et al., 2001; del Rio et al., 2010; Zhang and Bevan, 2013). It has a relevant role on cytotoxic function of CD8+ T cells in mucosae and against tumor cells, where it would bind E-cadherin at the immune synapse formed with target cells, allowing cytotoxic granules polarization (Le Floc'h et al., 2007; Smyth et al., 2007). CD103+CD8+ T cells are also relevant as lung-resident memory T cells, wherein they are helped by CD4+ T cells during influenza viral infection to confer protection against infection (Laidlaw et al., 2014). The KLGR receptor in NK cells engages E-cadherin and delivers a negative signal to the immune synapse formed by cytotoxic NK (Ito et al., 2006). KLGR1 expression in CD8 T cells also affects T cell effector cytolytic function and proliferation (Grundemann et al., 2006); CD8+ T cells expressing KLGR1 represent a subset of short-lived effector CD8+ T cells that do not develop into memory T cells (Joshi et al., 2007). KLGR1 recognizes an N-terminal homodimeric interface in E-cadherin and may interact with E-cadherin as a monomer to inhibit the immune response (Nakamura et al., 2009). The site of interaction of KLRG1 on E-cadherin is different from the mapped binding site for αEβ7 (Li et al., 2009), allowing dual interaction of E-cadherin with KLGR1 and αEβ7. These combinations of cis and trans molecular bonds may determine the organization of cell clusters at sites of interest between tumor cells, DCs, NKs, and T cells.

Therefore, different integrin receptors mediate interaction of T cells with APCs, probably facilitating simultaneous interaction with different cells and allowing stable interactions that help the effector function of T cells in different contexts. Integrins whose ligands are extracellular matrix proteins, such as αv integrins, can also have a role in the retention of immune cells at relevant sites, allowing mounting immune responses. In this regard, it is clear that integrin targeting has been successful in immunotherapies against autoimmune diseases with excessive recruitment of lymphocytes to tissues, but not in other diseases, such as cancer (Vicente-Manzanares and Sanchez-Madrid, 2018). In general, cancer anti-adhesive immunotherapies have been unsuccessful in contrast with those targeting checkpoint inhibitors such as PD-1 and CTLA-4, which improve sustained T cell activation and show great improvement in some malignant tumors, such as metastatic melanoma (Ribas and Wolchok, 2018). On one hand, cancer cells may escape from antibodies against specific integrins by expressing different integrins or using other adhesive molecules for movement during metastasis (Vicente-Manzanares and Sanchez-Madrid, 2018). On the other hand, it is possible that these anti-adhesive therapies, such as anti-αv integrins, which bind to extracellular matrix proteins as fibronectin, tenascin, or vitronectin and are expressed by CD4 and CD8 T cells (e.g., αvβ3), affect the quality of the immune synapses established in the tumor microenvironment, thereby preventing immune protection. Also, when targeting PD-1 and CTLA-4, adverse autoimmune side effects often occur. It is therefore essential to develop combined strategies that allow the control of the synapses established at the local sites of interest, allowing normal interaction of non-involved immune cells.

## Novel partners in cell-cell communication at the immune synapse

IL-7R and Notch1 receptors (thereafter Notch) are relevant mediators of T cell survival during development, based on their modulation of metabolic pathways (Waickman and Powell, 2012). Notch binds to its ligands in *trans*, Jagged or Delta, which induce Notch endocytosis and activation (Kopan and Ilagan, 2009). Notch segregation at the SMACs has been reported in alloreactive helper T (Th) cells in contact with DCs; Notch locates at cSMAC in Th cells whereas its inhibitor, Numb, appears in the plasma membrane of the pSMAC as well as in subcortical Rab11+ endosomes together with Notch ligands. The DC side mirrors this organization to allow signaling from the cSMAC in the Th and from the pSMAC in the DC (Luty et al., 2007). This is complementary to Notch activation upon TCR engagement in the absence of ligand. TCR activation promotes endocytosis of Notch, which is cleaved by ADAM upon PKC mediation (Steinbuck et al., 2018). In addition to its canonical, transcriptional function, Notch can also increase the activation of PI3K pathways upon TCR/CD28 co-stimulation of naïve CD4 T cells (Laky et al., 2015). In other cell types, such as endothelial cells, Notch activates eNOS activity (Villegas et al., 2018). It is conceivable that Notch modulates TCR-dependent signals by modifying the AKT/PI3K pathway, activates eNOS to promote actin reorganization, and modulates the relative localization of the TCR and LFA-1 molecules to organize the synapse.

## Concluding remarks

Integrins have been widely studied in T lymphocytes during development, migration and activation. However, it is not still entirely clear how they shape the signaling and form scaffold complexes that allow them to integrate extracellular and intracellular information. The relevance of their relationship with the TCR and chemokine receptors is a novel avenue of research that will unveil new connections in the ever-growing network of molecular connections at the T cell interface with the antigen-presenting cell. It is likely that this network will be mirrored by the corresponding ligands on the APC, which will determine the shape and dynamics of the adhesive contacts between the T cell and the APC, and will control the type of cell-cell communication to transmit information in a reciprocal manner. The control of diffusible second messengers, such as Ca^2+^ and nitric oxide at the IS will be of relevance for the establishment and regulation of polarity. The fine-tuning of the actin and tubulin cytoskeletons by the immune synapse will determine the ultimate fate of the T cell. All these events will influence the ability of the different immune cells to establish immune synapses leading to correct immune surveillance.

## Author contributions

NM-C wrote the manuscript and composed the figure. MV-M and FS-M wrote the manuscript.

### Conflict of interest statement

The authors declare that the research was conducted in the absence of any commercial or financial relationships that could be construed as a potential conflict of interest.

## Abbreviations

AKAP450: A kinase-anchoring protein of 450 kDa
AKT: RAC-alpha serine/threonine-protein kinase (PKB; protein kinase B)
APC: antigen-presenting cell
CD: cluster of differentiation
CRAC: calcium-release calcium channel
CTLA-4: cytotoxic T-lymphocyte-associated protein 4
DAG: diacylglycerol
DC: dendritic cell
eNOS: endothelial nitric oxide synthase
F-actin: filamentous actin
ICAM: intercellular adhesion molecule
GEF: GTP Exchange Factor
IP_3_: inositol-1,4,5-trisphosphate
IS: immune synapse
Jak: Janus Kinase
KLGR: killer cell lectin-like receptor G1
LAD: Leukocyte Adhesion Deficiency
LAT: linker for activation of T cells
Lck: Leukocyte C-terminal Src Kinase
LFA-1: Lymphocyte function-associated antigen-1
MHC: major histocompatibility complex
NMII-A: non-muscle myosin IIA
Orai: Calcium release-activated calcium channel protein 1
PD-1: programmed cell death-1
PI3K: phosphoinositide-3-kinase
PIP_2_: phosphatidylinositol-3,4-bisphosphate
PKC: protein kinase C
PLC: phospholipase C
ROCK: Rho-associated protein kinase
SMAC: supra-molecular activation cluster
STIM: Stromal interaction molecule
TCR: T cell receptor
VLA-4: Very Late Antigen-4
ZAP70: z-chain associated protein of 70 kDa.
